# Significant geographical differences in prevalence of mutations associated with *Plasmodium falciparum* and *Plasmodiumvivax* drug resistance in two regions from Papua New Guinea

**DOI:** 10.1186/s12936-015-0879-9

**Published:** 2015-10-09

**Authors:** Céline Barnadas, Lincoln Timinao, Sarah Javati, Jonah Iga, Elisheba Malau, Cristian Koepfli, Leanne J. Robinson, Nicolas Senn, Benson Kiniboro, Lawrence Rare, John C. Reeder, Peter M. Siba, Peter A. Zimmerman, Harin Karunajeewa, Timothy M. Davis, Ivo Mueller

**Affiliations:** Vector Borne Diseases Unit, Papua New Guinea Institute of Medical Research, Goroka, Papua New Guinea; Population Health and Immunity Division, Walter and Eliza Hall Institute of Medical Research, Parkville, Australia; Department of Medical Biology, University of Melbourne, Parkville, VIC Australia; Swiss Tropical and Public Health Institute, Basel, Switzerland; Burnet Institute, Melbourne, Australia; Center for Global Health and Diseases, Case Western Reserve University, Cleveland, USA; School of Medicine and Pharmacology, University of Western Australia, Perth, Australia; Centre de Recerca en Salut Internacional de Barcelona, Barcelona, Spain

**Keywords:** *Plasmodium vivax*, *Plasmodium falciparum*, Malaria, Drug resistance, Molecular markers, Papua New Guinea

## Abstract

**Background:**

Drug resistance remains a major obstacle to malaria treatment and control. It can arise and spread rapidly, and vary substantially even at sub-national level. National malaria programmes require cost-effective and timely ways of characterizing drug-resistance at multiple sites within their countries.

**Methods:**

An improved multiplexed post-PCR ligase detection reaction—fluorescent microsphere assay (LDR-FMA) was used to simultaneously determine the presence of mutations in chloroquine resistance transporter (*crt*), multidrug resistance 1 (*mdr1*), dihydrofolate reductase (*dhfr*) and dihydropteroate synthase (*dhps*) genes in *Plasmodium falciparum* (n = 727) and *Plasmodium vivax* (n = 574) isolates collected in 2006 from cross-sectional community population surveys in two geographically distinct regions (Madang and East Sepik) of Papua New Guinea (PNG) where strong regional differences in in vivo aminoquinoline and antifolate therapeutic efficacy had previously been observed. Data were compared to those of a follow-up survey conducted in 2010.

**Results:**

Despite some very low parasite densities, the assay successfully amplified all *P. falciparum* and *P. vivax* loci in 77 and 69 % of samples, respectively. In 2006, prevalences of *pfdhfr* (59R-108 N) double mutation/wild type *pfdhps* haplotype, *pfcrt* SVMNT haplotype (72S-76T double mutation), and 86Y *pfmdr1* mutation all exceeded 90 %. For *P. vivax*, 65 % carried at least two *pvdhfr* mutations, 97 % the 647P *pvdhps* mutation and 54 % the 976F *pvmdr1* mutation. Prevalence of mutant haplotypes was higher in Madang than East Sepik for *pfcrt* SVMNT (97.4 vs 83.3 %, p = 0.001), *pfdhfr* (59R-108 N) (100 vs 90.6 %, p = 0.001), *pvdhfr* haplotypes (75.8 vs 47.6 %, p = 0.001) and *pvmdr1* 976F (71.2 vs 26.2 %, p < 0.001). Data from a subsequent Madang survey in 2010 showed that the prevalence of *pfdhps* mutations increased significantly from <5 % to >30 % (p < 0.001) as did the prevalence of *pvdhfr* mutant haplotypes (from 75.8 to 97.4 %, p = 0.012).

**Conclusions:**

This LDR-FMA multiplex platform shows feasibility for low-cost, high-throughput, rapid characterization of a broad range of drug-resistance markers in low parasitaemia infections. Significant geographical differences in mutation prevalence correlate with previous genotyping surveys and in vivo trials and may reflect variable drug pressure and differences in health-care access in these two PNG populations.

**Electronic supplementary material:**

The online version of this article (doi:10.1186/s12936-015-0879-9) contains supplementary material, which is available to authorized users.

## Background

Monitoring the emergence and spread of drug resistance is a key priority for malaria control and elimination efforts in the modern era [1]. The recent emergence of artemisinin resistance in South-east Asia demonstrates that drug resistance can spread rapidly across national borders and how its prevalence can vary substantially even within individual countries [2]. Effective containment strategies will require comprehensive, accurate, real-time estimates of drug resistance status from multiple sites that can identify the potential for treatment failure within small geographic regions.

In vivo evaluations of drug susceptibility are expensive and logistically challenging, requiring a substantial time-commitment from participants and appropriate clinical research infrastructure at each study site. Sample size constraints and the confounding effects of local transmission (risk of re-infection) during follow-up can compromise comparisons between different geographic locations. In vivo evaluations are, therefore, inappropriate for rapid and comprehensive comparisons of drug resistance between distinct sites within a single country or small geographic area.

The recent development of well-characterized molecular markers that correlate strongly with in vivo drug resistance has meant that there are now feasible alternative in vitro methods of characterizing population drug resistance. Resistance to sulfadoxine-pyrimethamine (SP) in *Plasmodium falciparum* is clearly linked to mutations in the dihydrofolate reductase (*pfdhfr)* and dihydropteroate synthase (*pfdhps*) genes with selection thought to occur as a step-wise process leading to highly resistant triple to quintuple mutant parasites [3]. There are orthologs of these genes in *P. vivax* [4–8], and *pvdhfr* mutations at codons 57, 58, 61, 117, 173 correlate with SP resistance [9, 10]. *P. falciparum* 4-aminoquinoline resistance correlates with mutations in *pfcrt* [11] and *pfmdr1.* However, the relationship between 4-aminoquinoline resistance and mutations in the *P. vivax* ortholog *(pvmdr1*) remains controversial [12–14] even though it seems that a codon 976 mutation is associated with AQ or CQ drug resistance in the Asia–Pacific region [13].

Of the four *Plasmodium* species present in Papua New Guinea (PNG), *P. falciparum* and *P. vivax* predominate [15, 16]. The prevalence of each species differs according to the geography of the country and among age groups [17]. This complex epidemiology makes malaria control in PNG very challenging. Early malaria control efforts in PNG included mass drug administration (MDA) in the 1950s and 1960s [18, 19]. Employed variably throughout the country, some areas received MDA with chloroquine, some with pyrimethamine and others no drug treatment at all [20]. Anti-malarial treatment policy for case management was chloroquine (CQ) for adults or amodiaquine (AQ) for young children until 2000 [21]. However, *P. falciparum* CQ resistance had started to emerge as early as the 1970s [22] and in vivo resistance rates as high as 47 % were documented in Madang Province by the early 1980s [20]. Resistance in *P. vivax* emerged much later, with the first well-documented CQ treatment failure described in 1989 [23, 24] and remained at 4.5 % in East Sepik by 2000 [25].

In 2000, PNG treatment policy was changed to incorporate SP as first-line therapy in combination with AQ or CQ. At around that time, *P. falciparum* PCR-corrected failure rates ranged from 18.2 to 28.6 % for CQ-SP and 10.3 to 21.5 % for AQ-SP in different regions [26]. For *P. vivax,* estimates of resistance to AQ/CQ-SP ranged from 0 to 7 % in the same provinces [26] whilst uncorrected treatment failure rates of 49 % (28 days) and 87 % (42 days) were documented in Madang and Sepik in 2005–2006 [27]. However, in contrast to *P. falciparum*, the absence of validated markers to distinguish recrudescence from reinfection have meant that in vivo comparisons of *P. vivax* therapeutic efficacy between sites with different malaria epidemiology are confounded by both new infections and hypnozoite relapses during follow-up, especially in areas of high transmission [28].

Following observations of heterogeneity of in vivo drug resistance in Madang and East Sepik provinces of PNG [26, 29], the utility of an established ligase detection reaction fluorescence microsphere assay (LDR-FMA) was investigated [29]. A novel primer set was multiplexed to characterize mutations associated with SP and 4-aminoquinoline resistance in both *P. falciparum* and *P. vivax* from cross-sectional population surveys conducted in these two provinces. The aim of the study was to demonstrate the feasibility of this high-throughput technology as a practical and cost-effective means of rapidly comparing susceptibility to multiple drugs at numerous geographic sites.

## Methods

### Study population and blood sample collection

Cross-sectional population-wide surveys were conducted in Maprik, East Sepik Province (2006) and Mugil, Madang Province (2006 and 2010). Following informed consent from adults or children’s guardians, 200 μL of whole blood was collected by fingerprick from participants of all ages. The study was approved by the Medical Research Advisory Committee (#05-20) of PNG and the PNG Institute of Medical Research Institutional Review Board (#06.01).

### DNA extraction and molecular diagnosis of *Plasmodium species* infections

Genomic DNA was extracted from whole blood using the QIAamp 96 DNA blood kit (Qiagen, Valencia, CA) or the 96-well genomic DNA extraction kit (Favorgen, Taiwan) according to the manufacturer instructions. The presence of *P. falciparum* and *P. vivax* infections was determined by a post-PCR, ligation detection reaction—fluorescent microsphere assay (LDR-FMA: 2006 surveys) [30] and a qPCR assay (2010 survey) [31, 32].

### Detection of mutations in *Plasmodium falciparum dhfr*, *dhps* and *mdr1* genes

Detection of mutations in *pfdhfr, pfdhps, pfmdr1* and *pfcrt* was achieved by LDR-FMA using an adaptation of the methods of Carnevale et al. [33] and Wong et al. [34]. New primers were designed in order to multiplex the PCR and perform nested PCR. Primer sequences are provided in Additional file 1: Table S1. All PCR-1 reactions were performed in a buffer containing 120 nM of each primer, 67 mM Tris–HCl (pH = 8.8), 6.7 mM MgSO_4_, 16.6 mM (NH_4_)_2_SO_4_, 10 mM mercaptoethanol, 200 µM (each) dATP, dGTP, dCTP, dTTP, and 2.5 U of thermostable DNA polymerase. Nested PCR-2 reactions were performed with 3 µL of PCR products from the PCR-1 reaction using the same conditions except for the primers (240 nM each). PCR amplification was performed under the following conditions: PCR-1—95 °C for 2 min and 30 cycles at 95 °C for 30 s, 52 °C for 30 s, 72 °C for 80 s, and a final extension at 72 °C for 5 min; PCR-2—95 °C for 2 min and 40 cycles at 95 °C for 30 s, 59 °C for 30 s, 72 °C for 1 min, and a final extension at 72 °C for 5 min. Amplification specificity was evaluated following electrophoresis on 2 % agarose gel. In order to integrate detection of mutations in *pfmdr1*, new LDR primers were designed (sequences provided in Additional file 2: Table S2). LDR-FMA was performed with 1 μL of PCR-2 product with cycling conditions and fluorescence signal interpretation as described previously [29, 35].

Because a high background signal for the wild type *pfmdr* 86 allele raised concerns regarding the LDR primer’s specificity in polyclonal infections, a PCR RFLP protocol was used to confirm LDR-FMA genotyping results. PCRs were performed using the same primer sets as the LDR-FMA. RFLP was achieved by mixing 5 μL of PCR products with 15 μL of a mix containing 1 U of ApoI restriction enzyme (New England Biolabs, USA), 1X NE buffer and 1X BSA. The digestion took place at 50 °C for 2 h. Digested products were visualized on 2 % agarose gel.

### Detection of mutations in *Plasmodium vivax dhfr*, *dhps* and *mdr1* genes

Detection of mutations in *pvdhfr, pvdhps* and *pvmdr1* gene was achieved using a LDR-FMA as described previously [29]. Two LDR allele-specific primers were added to the 23 primers already used in the assay in order to integrate two *pvdhfr* alleles newly identified in PNG by sequencing (Additional file 2: Table S2). Fluorescence signals were interpreted as previously described [28, 35].

### Statistics

Chi square and Fisher exact tests (Stata 12) were used to compare the frequency of *P. falciparum* and *P. vivax* infections and mutation prevalence between East Sepik and Madang populations.

## Results

### Study population and *Plasmodium* infections

As previously detailed [36], a total of 2359 samples, including 1282 (54.3 %) from Madang and 1077 (45.7 %) from East Sepik, were collected in 2006. Of these, 784 (61.1 %) samples from Madang and 416 (38.9 %) from East Sepik had PCR-detected *Plasmodium spp* (p < 0.001). *Plasmodium falciparum* was present in 487 (62.1 %) and 240 (57.7 %) (p = 0.14, NS) and *P. vivax* in 409 (52.2 %) and 165 (39.7 %) of infections (p < 0.001) from Madang and East Sepik, respectively. A further 88 *P. falciparum* and 89 *P. vivax* positive samples collected in a 2010 survey in Madang were also selected for drug resistance genotyping.

### Drug resistance genotyping reveals geographic heterogeneity of parasite populations

#### *Plasmodium falciparum* genotyping

Of 399 randomly-selected 2006 samples, genotyping was successfully performed on the four genes (*pfdhfr, pfdhps, pfcrt* and *pfmdr1*) in 307 (76.9. %). An additional 28 samples (7.1 %) yielded results for at least one of the four genes. Mutations in *pfdhfr, pfdhps* and *pfmdr1* were detected individually except for codons 436–437 of *pfdhps*. Single gene and multigenic haplotypes were only determined in participants displaying a monoclonal infection as determined by the maximum number of alleles (n = 1) detected at *pfdhfr, pfdhps, pfcrt* and *pfmdr1*. Genotyping results are displayed in Table 1. Over 95 % of isolates displayed a double mutation at *pfdhfr* (59R-108N) and a wild type haplotype at *pfdhps*. A small number (7 samples: 4.8 %) had *pfdhps* mutations in addition to *pfdhfr* mutations, and thus carried triple or quadruple mutant *pfdhfr/pfdhps* haplotypes.

**Table 1:** *Pfdhfr, pfdhps, pfcrt* and *pfmdr1* haplotypes among *P. falciparum* monoclonal infections

	Total 2006 (%)	East Sepik 2006 (%)	Madang 2006 (%)	p value	Madang 2010 (%)	p value
Monoclonal infections	210	96	114		63	
*pfdhfr* genotype (codons 51-59-108-164)						
Wild type						
N-C-S-I	5 (2.4)	5 (5.2)	0	0.001	0	NS
Single mutant						
N-C-**N**-I	3 (1.4)	3 (3.1)	0		0	
N-**R**-S-I	0	0	0		1 (1.6)	
Double mutant						
**I**-C-**N**-I	1 (0.5)	1 (1.0)	0		0	
N-**R**-**N**-I	201 (95.7)	87 (90.6)	114 (100)		62 (98.4)	
*pfdhps* genotype (codons 436/437-540-581-613)						
Wild type:						
SA-K-A-A	200 (95.2)	89 (92.7)	110 (96.5)	NS	43 (68.3)	<0.001
Single mutant:						
S**G**-K-A-A	7 (3.3)	3 (3.1)	4 (3.5)		10 (15.9)	
Double mutant						
S**G**-**E**-A-A	2 (1.0)	2 (2.1)	0		10 (15.9)	
S**G**-K-**G**-A	1 (0.5)	1 (1.0)	0		0	
*pfcrt* genotype (codons 72/73/74/75/76)						
Wild type						
CVMNK	18 (8.6)	15 (15.6)	3 (2.7)	0.001	1 (1.2)	NS
Double mutant						
**S**VMN**T**	191 (91.0)	80 (83.3)	111 (97.4)		62 (98.4)	
Triple mutant						
CV**IET**	1 (0.5)	1 (1.0)	0		0	
*pfmdr1* genotype (codons 86-184-1034-1042-1246)						
Wild type						
N-Y-S-N-D	10 (4.8)	6 (6.3)	4 (3.5)	NS	0	NS
Single mutant						
**Y**-Y-S-N-D	196 (93.3)	88 (91.7)	108 (94.7)		62 (98.4)	
Double mutant						
N-**F**-S-**D**-D	2 (1.0)	0	2 (1.8)		0	
**Y**-Y-S-**D**-D	1 (0.5)	1 (1.0)	0		0	
Triple mutant						
**Y**-**F**-S-**D**-D	0	0	0		1 (1.2)	
Quintuple mutant						
**Y**-**F**-**C**-**D**-**Y**	1 (0.5)	1 (1.0)	0		0	

Most isolates (>90 %) displayed the *pfcrt* SVMNT haplotype (i.e. a double 72S-76T mutations) and a *pfmdr1 *single mutation (86Y). Only one East Sepik sample carried the triple mutation (74I-75E-76T) at *pfcrt* (CVIET haplotype). Despite the high overall frequency of *pfcrt* SVMNT and *pfdhfr* double mutant haplotypes, their prevalence was significantly higher in Madang than in East Sepik (Table 1: p = 0.001). These results were confirmed when looking at the prevalence of mutations in all samples (monoclonal and polyclonal infections). Wild-type *pfdhfr* alleles (codons 59 and 108) were more frequent in East Sepik than Madang (p < 0.001; Additional file 3: Table S3) as was wild-type CVMNK *pfcrt* haplotype (p < 0.001). However *pfmdr1* mutations at codons 184 and 1042 were more often observed in East Sepik (p = 0.005 and p = 0.03). Both resistant CVIET and SVMNT alleles were found in East Sepik.

### *Plasmodium vivax* genotyping

Genotyping was successfully performed on the three genes (*pvdhfr, pvdhps* and *pvmdr1*) in 398 (69.3 %) of 574 *P. vivax* LDR-FMA samples and at least one of the three genes in 483 (84.1 %). As described previously [29], mutations in *pvdhfr, pvdhps* and *pvmdr1* were detected either individually (*pvdhfr* codons 117, 173; *pvdhps* codons 553, 647; *pvmdr1* codon 976), or as a haplotype (*pvdhfr* codons 57-58-61; *pvdhps* codons 382–383). For this reason, while it was possible to affirm the presence of triple mutant isolates at *pvdhfr* codons 57-58-61, it was only possible to confirm the presence of quadruple mutant haplotypes (triple mutant at codons 57-58-61 plus mutant at codon 117) in participants with monoclonal infections. Genotyping results are displayed in Table 2.

**Table 2:** *Pvdhfr, pvdhps* and *pvmdr1* haplotypes among *P. vivax* monoclonal infections

	Total 2006 (%)	East Sepik 2006 (%)	Madang 2006 (%)	p value	Madang 2010 (%)	p value
Monoclonal infections	108	42	66		38	
*pvdhfr* genotype (codons 57/58/61-117-173)						
Wild type						
FST-S-I	38 (35.2)	22 (52.4)	16 (24.2)	0.001	1 (2.6)	0.012
Single mutant						
FST-**N**-I	1 (0.9)	1 (2.4)	0		0	
Double mutant						
F**R**T-**N**-I	4 (3.7)	1 (2.4)	3 (4.5)		1 (2.6)	
**LR**T-S-I	22 (20.4)	10 (23.8)	12 (18.2)		7 (18.4)	
Triple mutant						
**LR**T-**T**-I	2 (1.9)	1 (2.4)	1 (1.5)		3 (7.9)	
Quadruple mutant						
**LRM**-**T**-I	41 (38.0)	7 (16.7)	34 (51.5)		26 (68.4)	
*pvdhps* genotype (codons 382/383-553-647)						
Wild type						
SC-A-A	3 (2.8)	0	3 (4.5)	NS	4 (10.5)	NS
Single mutant						
SC-A-**P**	104 (96.3)	42 (100)	62 (93.9)		34 (89.5)	
Double mutant						
S**G**-A-**P**	1 (0.9)	0	1 (1.5)		0	
*pvmdr1* genotype (codon 976)						
Wild type						
Y	50 (46.3)	31 (73.8)	19 (28.8)	<0.001	11 (28.9)	NS
Mutant						
**F**	58 (53.7)	11 (26.2)	47 (71.2)		27 (71.1)	

Only 38 of 108 monoclonal isolates (35.2 %) displayed a wild type haplotype at *pvdhfr* with 26 (24.1 %) and 41 (38.0 %) displaying double and quadruple mutant haplotypes, respectively. The vast majority of the isolates (97.2 %) carried the 647P mutation in *pvdhps* and 58 (53.7 %) the 976F mutation in *pvmdr1*. The *pvmdr1* 976 mutation itself was highly significantly associated with the *pvdfhr* quadruple mutant haplotype (OR = 5.6; CI_95_ = [2.3;13.6], p < 0.001). The prevalence of mutant *pvdhfr* haplotypes was significantly higher in Madang than East Sepik (75.8 vs 47.6 %, p = 0.001), with prevalence of the quadruple mutant haplotype reaching 51.5 % in Madang vs 16.7 % in East Sepik. Similarly, *pvmdr1* 976F mutation prevalence was higher in Madang than East Sepik (71.2 vs 26.2 %, p < 0.001).

These results were confirmed when including all infections (monoclonal and polyclonal) (Additional file 4: Table S4). Two additional mutations were observed but at low frequency: *pvdhfr* codon 173 (I → L; 0.4 %) and *pvdhps* codon 647 (A → S; 0.2 %).

### Selection of additional mutant genotypes under continuous drug pressure

Genotyping results of *P. falciparum* and *P. vivax* isolates collected in 2006 in Madang Province were compared with samples collected at the same site in 2010 (Tables 1, 2). As mutant *pfdhfr* and *pfmdr1* genotypes had already nearly reached fixation in Madang in 2006, no significant change was observed in 2010. The prevalence of the *pfcrt* K76 wild type allele decreased from 13.6 to 1.2 % (p = 0.002, Additional file 3: Table S3). A dramatic increase in the prevalence of *pfdhps* mutations was observed. In 2010, 15.9 % of isolates carried a single mutation (437 G, vs 3.5 % in 2006, p = 0.004) and another 15.9 % a double mutation (437G/581G, vs 0 % in 2006, p < 0.001). For *P. vivax*, while the prevalence of mutant *pvdhps* and *pvmdr1* haplotypes remained stable over time, a significant increase was observed for *pvdhfr,* (97.4 % in 2010 vs 75.8 % in 2006, p = 0.005).

## Discussion

The present study shows that the highly multiplexed LDR-FMA platform performed well in characterizing multiple loci related to 4-aminoquinoline and antifolate drug resistance in both *P. falciparum* and *P. vivax.* Through its successful application in low parasite density infections (from cross-sectional population surveys of mostly asymptomatic individuals) and by generating results highly concordant with existing in vivo drug resistance data (reflecting both spatial and temporal differences in population drug resistance profiles), the LDR-FMA platform can be used for rapid, low-cost application at multiple field sites. This makes it an attractive tool for national malaria programmes that require comprehensive local level characterization of drug resistance patterns.

Existing LDR-FMA assays [33, 34] were significantly improved by using new PCR primers designed to allow visualization of the five amplified fragments of *pfmdr1*, *pfcrt, pfdhfr* and *pfdhps* on an agarose gel in a single multiplexed assay. Compared with previously published LDR-FMA protocols, the new assay requires much less total DNA, less technician time and has lower overall assay costs. Even though the assay was performed in samples from mostly asymptomatic individuals who a had much lower parasitaemia than previous studies utilizing samples from patients with symptomatic infections [34], it was only slightly less sensitive for *P. vivax* and equivalent for *P. falciparum* [29, 34]. Multiplexing the PCR in a nested PCR protocol did not, therefore, compromise assay performance significantly. A potential problem in the *P. falciparum* component of the assay related to high background signal for the wild type *pfmdr* 86 allele (that raised concerns regarding the LDR primer’s specificity in polyclonal infections) was addressed using a PCR RFLP protocol. The *P. vivax* assay was also improved by designing an allele specific primer targeting a new polymorphism in the *pvdhfr* codon 57.

The prevalence of the *P. falciparum* 4-aminoquinoline resistance mutations reported here is very similar to previously published data including a >80 % prevalence of *pfmdr1* 86Y in 2000–2005 [37–39] and 86–96 % prevalence of *pfcrt* 76T mutation in the early 2000s in East Sepik and Madang [33, 37–40]. The *pfcrt* 76T mutation is almost exclusively found as part of the double mutant haplotype **S**VMN**T** previously described in PNG and South America [41, 42]. While this mutation has been reaching levels near fixation (96 %) in symptomatic infections from children from the same geographical area (Mugil village, Madang Province) over the last 9 years [43], a similar prevalence is observed here in asymptomatic individuals of all ages.

A recent analysis by Nsanzabana et al. has suggested that strong selection of *pfdhfr* 59R 108N double mutant parasites occurred in East Sepik in the 3 years following the introduction of SP in the early 2000s, with a 2.5 times increase in double mutant prevalence to 60–70 % over this time [44]. This has progressed with levels approaching fixation in the 2006 surveys and other data from the same provinces in 2005–2007 [34]. By contrast, the frequency of *pfdhps* mutations remained very low in 2006 suggesting some retention of sulfadoxine activity. However, the emergence of significant rates of single and double mutant *pfdhps* in the 2010 data (combined prevalence of 31.8 %) suggests that a loss of SP efficacy may be imminent in Madang Province, thus threatening its potential value for intermittent preventive treatment in pregnancy and infants.

Despite the very high overall frequency of many mutations in the present study, at least four mutations (*pfcrt*, *pfdhfr, pvdhfr* haplotypes and the *pvmdr1* 976F) were significantly more prevalent in Madang compared with East Sepik, which is in accord with similar observations of differences in drug resistance mutations between the two areas [37]. One explanation for this phenomenon is that the Madang region has more functional health-care infrastructure and, therefore, its population has better access to anti-malarial drugs than the more remote populations of the East Sepik [44], resulting in a higher selection pressure. Distribution of drugs beyond their expiration date leading to sub optimal dosage could also have contributed to the selection of drug resistant parasites.

The significant differences in *pvmdr1* 976F mutation prevalence between the two provinces (72 vs 26 % in monoclonal infections) and similar differences observed for *pvdhfr* mutant genotypes accord well with the observations from in vivo studies of SP + CQ or AQ efficacy of *P. vivax* infections between 2003 and 2005 that demonstrated no treatment failure in East Sepik but 29 % treatment failures in Madang [26]. Overall, the quadruple *pvdhfr* genotype was identified in 38.5 % of the *P. vivax* monoclonal infections. This genotype, when found in combination with a mutation at *pvmdr1* 976, has previously been associated with AQ-SP treatment failure in a study conducted in PNG children [9]. Apart from the 647**P** mutation that seems to be fixed in the PNG *P. vivax* population, mutations in *pvdhps* were rarely found.

*Plasmodium vivax* is characterized by early production of gametocytes during its life cycle (prior to the first symptoms of malaria) so that transmission can occur prior to anti-malarial drug exposure, therefore delaying the selection of drug resistant isolates. However, in PNG, the high incidence of mixed *P. falciparum*/*P. vivax* infections may result in an early exposure of concurrent *P. vivax* infections to anti-malarial drugs at the time of treatment of symptomatic malaria caused by *P. falciparum*. Additionally, *P. vivax* polyclonal infections are very frequent with up to 75 % of all infected carrying several genotypes [45]. This leads to numerous opportunities for sexual recombination between parasite clones during the mosquito phase of the parasite life cycle and therefore production of meiotic recombinant parasites with drug resistant genotypes. Despite a lower *P. vivax* prevalence in East Sepik than in Madang, both parasite populations have shown similar levels of genetic diversity [36]. Therefore, as with observations of geographic differences in *P. falciparum* drug resistance mutants, the higher prevalence of *pvmdr1* and *pvdhfr* mutants in the Madang *P. vivax* population may reflect greater drug pressure in the Madang area.

Like most malaria-endemic countries, PNG has now adopted WHO recommended artemisinin combination therapy (ACT) as the cornerstone of its anti-malarial case-management strategy. This change to ACT has already been associated with significant reductions in malaria prevalence elsewhere but the emergence and spread of artemisinin resistance in South-East Asia [2], and observations of variable response to partner drugs (such as lumefantrine) [46] suggest the need for ongoing surveillance of drug susceptibility to both components. Recent identification of an association of particular single nucleotide polymorphisms in the *kelch*-*13* gene with artemisinin resistant phenotypes [47] may enable incorporation of markers of artemisinin resistance in combination with those evaluated in this study. As demonstrated here, the LDR-FMA platform is highly flexible and integration of new markers can easily be achieved.

It is also notable that lumefantrine exerts a selective pressure on *pfmdr1* N86Y in an opposing direction to that of AQ and CQ [46, 48, 49]. Therefore, reversion to wild-type genotypes following uptake of artemether-lumefantrine may indicate diminishing parasite susceptibility to lumefantrine but may also raise the prospect of resurrecting AQ or CQ as effective treatments.

## Conclusion

Significant geographical differences in mutation prevalence in the present study correlate with recent in vivo data obtained from the same regions and may reflect variable drug pressure due to differences in health-care access in the two PNG populations. The high-throughput capacity and relatively low cost of the LDR-FMA assay could make it a valuable tool for rapidly characterizing in a central laboratory the status of resistance to a range of anti-malarial drugs from sites that may not necessarily have the infrastructure required for conducting in vivo evaluations of drug efficacy. This can be achieved by National Malaria Control Programmes and partner organizations using isolates obtained through cross-sectional population sampling.

## Additional files

10.1186/s12936-015-0879-9 Sequences of PCR primers.
10.1186/s12936-015-0879-9 Sequences of new LDR primers.
10.1186/s12936-015-0879-9 *Pfdhfr*, *pfdhps*, *pfcrt* and *pfmdr1* genotypes prevalence in all *P. falciparum* infections.
10.1186/s12936-015-0879-9 *Pvdhfr*, *pvdhps* and *pvmdr1* genotypes prevalence in all *P. vivax* infections.

## Abbreviations

ACT: artemisinin combination therapy
AQ: amodiaquine
crt: chloroquine resistance gene
CQ: chloroquine
dhfr: dihydrofolate reducatse gene
dhps: dihydropteroate synthase gene
LDR-FMA: ligase detection reaction—fluorescent microsphere assay
MDA: mass drug administration
mdr1: multidrug resistance 1 gene
PNG: Papua New Guinea
SP: sulfadoxine-pyrimethamine
